# Informative triage scales may be superior to non-informative ones in case of poor clinical expertise of raters

**DOI:** 10.1186/s12873-023-00817-7

**Published:** 2023-05-15

**Authors:** Amir Mirhaghi

**Affiliations:** Nursing and Midwifery Care Research Center, Mashhad University of Medical Sciences, Mashhad, Iran

Dear editor

The manuscript titled “*FRENCH versus ESI: comparison between two nurse triage emergency scales with referent scenarios*”, by Aubrion et al., was reviewed with great interest. This study is to initially compare the validity of the Emergency Severity Index (ESI), as a triage tool, and the French Emergency Nurses Classification in-Hospital (FRENCH) scale, with reference to a number of standard triage scenarios. It also aims to determine the interrater reproducibility of triage decision-making among the nurses recruited. The study results had accordingly revealed that the FRENCH scale had shown higher validity with better outcomes, as compared with the ESI, wherein the elevated rates of undertriage had been reported in critically ill patients [1]. In addition, no significant difference had been observed between both scales with respect to the reproducibility between the raters (the nurses and nursing students). In view of that, some key points here called for further explanation.

The first point regarding the ESI validity is that several studies have reported overtriage is the major mistriage in ESI [2, 3], although it is reported that undertriage will also happen [4]. In a study with a total of 5,315,176 ED encounters, mistriage occurred in 32.2% encounters, of which 3.3% were undertriaged and 28.9% were overtriaged. Besides, overtriage in the ESI scale is addressed in the ESI implementation book 2020 “*Triage nurses without sufficient ED experience may be at risk for overtriaging patients.*” [5]. Criteria in Level I and II of ESI scale may be ambiguous for less experienced nurses. For example, the decision criteria at Level 1 is: “Does the patient require immediate, life-saving interventions?“ and that at Level 2 is “Is the patient in a high-risk situation, confused, lethargic, or disoriented, or suffering from severe pain or distress?“. The major point is that all situations at Levels 1 and 2 naturally seem subjective; and substantial clinical expertise is needed to differentiate these situations from others. High risk situation is main cause of overtriage in ESI scale, because many novice nurses may assume patient’s condition as a high risk situation in uncertain conditions. Uncertainty is reported in triage studies [6]. Accordingly, nurses might assign patients to a higher category and cause overtriage, once dealt with uncertain situations. Overtriage has been further reported to be between 13.6% and 31%, especially in patients presenting with chest pain or dyspnea [4, 7].

In this study, the nurses and nursing students had been subjected to overtriage by 6.7% and 8.5% at ESI Level 2, which raises the question of how this happened. Additionally, undertriage at ESI Level 2 was 50% in the nurses and 48% in the nursing students, which were respectively similar to 43% and 62% in the nurses and nursing students at ESI Level 1. The questions addressed here were why these raters, showed more undertriage than overtriage based on the ESI, and even why the nursing students committed mistriage more than the nurses. The main reason for the biases in the study results here was that the using the ESI could be influenced by the clinical expertise of the raters. Clinical judgments among nurses, as a confounding variable, could also moderate the validity of the results. Hence, internal validity might have been biased by the poor clinical expertise among the nursing students.

These findings are reinforced by observing the results at Levels 4 and 5, in which the undertriage rates by the nurses and nursing students, using the ESI, were 43% and 21%, respectively. The overtriage values in the nurses were also 7% and 34%, and they were equal to 23% and 55% among the nursing students. Differentiating the categories of triage at Levels 4 and 5 with reference to the ESI accordingly requires that the triage nurses have the ability to predict the number of resources for the patients during hospitalization, and actually reach an initial diagnosis, which further demands clinical expertise. Here, the nursing students’ mistriage was higher than that in the nurses, indicating that the study results might have been biased by the poor clinical judgments of the assessors, because decision-making based on the ESI is strongly affected by the clinical expertise of the triage nurses.

The question raised here was why the validity outcomes of the FRENCH scale were better than those in the ESI. Notably, a number of scales such as the Manchester Triage System (MTS) and the FRENCH scale, which are specialized tools developed for major complaints, are typically associated with better validity, particularly when raters do not have expertise in terms of clinical judgment. The scales that explain chief complaints are thus more informative and assist raters. Hence, such scales can probably provide better validity. In this respect, the mean unweighted value of kappa in regard to reference was 0.41 (namely, moderate) in the nurses (0.37–0.50) and 0.28 (viz., fair) in the nursing students (0.21–0.33), suggesting that the clinical expertise of the assessors was effective in the validity of the study results. Therefore, comparing FRENCH scale with ESI scale may not be appropriate in this study because of differences that is embedded in the nature of scales and not considering clinical expertise of raters.

Another important point to mention is that the structure of the scenarios could significantly affect the validity results. It has been further reported in some studies that the poor structure of scenarios can bias the reliability outcomes. The test-retest reliability results had accordingly reported the mean unweighted value of kappa of 0.33 (0.26–0.39) for the nurses and 0.18 (0.13–0.22) for the nursing students, respectively interpreted as fair and slight, which were not in line with previous research. Meta-analysis has further demonstrated that the ESI reliability is at the significance level of 0.791 (95% confidence interval [CI]: 0.787–0.795), which is much higher than these values [8]. The reliability of the given scales could also depend on structure of scenarios. If the scenarios include major complaints, vital signs and associated signs and symptoms, and demographic characteristics that are not defined, they cause the raters to evaluate the same scenario differently at various times, which results in poor reliability. Hence, it is of utmost importance to be very careful about the comprehensive definition of scenarios in triage reliability assessment research. As a whole, the readers are suggested to interpret the study results with regard to the cofounding effect of the raters` clinical expertise or the possible poorly structured scenarios. Therefore, no superiority of FRENCH scale over ESI scale should be assumed based on this study, but it can be said that informative triage scales may be superior to other non-informative scales in case of poor clinical expertise.

## Data Availability

Not applicable.
